# Therapeutic effects of rapamycin and surgical decompression in a rabbit spinal cord injury model

**DOI:** 10.1038/s41419-020-02767-5

**Published:** 2020-07-23

**Authors:** Xin Zhang, Chuan Qin, Yingli Jing, Degang Yang, Changbin Liu, Feng Gao, Chao Zhang, Zuliyaer Talifu, Mingliang Yang, Liangjie Du, Jianjun Li

**Affiliations:** 1 School of Rehabilitation Medicine, Capital Medical University, Beijing, 100068 China; 2 China Rehabilitation Science Institute, Beijing, 100068 China; 3 Center of Neural Injury and Repair, Beijing Institute for Brain Disorders, Beijing, 100068 China; 4 Department of Spinal and Neural Functional Reconstruction, China Rehabilitation Research Center, Beijing, 100068 China; 5 Beijing Key Laboratory of Neural Injury and Rehabilitation, Beijing, 100068 China; 6 Institute of Rehabilitation Medicine, China Rehabilitation Research Center, Beijing, 100068 China; 7 Department of Rehabilitation Medicine, Beijing Tiantan Hospital, Beijing, 100050 China

**Keywords:** Spinal cord injury, Neurophysiology, Spinal cord diseases

## Abstract

Surgical decompression after spinal cord injury (SCI) is a conventional treatment. Although it has been proven to have clinical effects, there are certain limitations, such as the surgical conditions that must be met and the invasive nature of the treatment. Therefore, there is an urgent need to develop a simple and maneuverable therapy for the emergency treatment of patients with SCI before surgery. Rapamycin (RAPA) has been reported to have potential as a therapeutic agent for SCI. In this study, we observed the therapeutic effects of rapamycin and surgical decompression, in combination or separately, on the histopathology in rabbits with SCI. After combination therapy, intramedullary pressure (IMP) decreased significantly, autophagic flux increased, and apoptosis and demyelination were significantly reduced. Compared with RAPA/surgical decompression alone, the combination therapy had a significantly better effect. In addition, we evaluated the effects of mechanical pressure on autophagy after SCI by assessing changes in autophagic initiation, degradation, and flux. Increased IMP after SCI inhibited autophagic degradation and impaired autophagic flux. Decompression improved autophagic flux after SCI. Our findings provide novel evidence of a promising strategy for the treatment of SCI in the future. The combination therapy may effectively improve emergency treatment after SCI and promote the therapeutic effect of decompression. This study also contributes to a better understanding of the effects of mechanical pressure on autophagy after neurotrauma.

## Introduction

Spinal cord injury (SCI) has become a major health issue of global concern. The global age-standardized prevalence of SCI in 2016 was 368 cases per million individuals, and the years of life lived with disability (YLD) was 9.5 million, which accounted for a considerable proportion of the global injury burden^1^. Even with the current major clinical interventions for patients, such as surgical decompression, stem cell therapy, neurotrophic factor treatment, and platelet-rich plasma treatment, nerve damage is difficult to repair.

Decompression after SCI is a conventional treatment method^2^ and has been proven to have significant beneficial effects. This method releases pressure on the swollen spinal cord, increases spinal blood perfusion, reduces secondary injuries, and improves nerve function^3–7^. However, due to limitations, not all patients can be treated correctly and in a timely manner after injury^8^, and invasive surgery can have certain side effects^9^. Therefore, there is an urgent need to develop a simple and feasible therapy for the emergency treatment of patients with SCI before surgery.

Rapamycin (RAPA) increases autophagy by inhibiting mTOR in mammals^10,11^. After SCI, RAPA intervention upregulates autophagy, inhibits apoptosis and the inflammatory response, and improves motor function^12–17^. This suggests that RAPA may have potential as a therapeutic agent in SCI. Therefore, we hypothesized that surgical decompression combined with RAPA may contribute to the treatment of SCI.

At present, it has been shown that stress-induced autophagy leads to increased Beclin-1 and LC3 expression^18,19^. Multiple studies have observed stress-related autophagy responses. For example, mechanical stress (strain, compression, shear stress, etc.) that induces autophagy^20,21^, compression forces that rapidly increases autophagosome numbers in cells^22^, and arteriovenous shear stress that causes excessive vascular endothelial cells autophagy and apoptosis^23^. Despite these findings, the role of intramedullary pressure (IMP) in autophagy after SCI is not known. Deeply understanding its potential mechanisms will be of great value for future attempts to manipulate IMP and autophagy as a therapeutic strategy.

The aim of this study was to investigate the therapeutic effects of rapamycin and surgical decompression, in combination or separately, on the histopathology of the injured spinal cord in a rabbit model of SCI. Furthermore, the effects of IMP on autophagy after SCI were explored in this study.

## Results

### RAPA combined with surgical decompression significantly reduced IMP and inhibited apoptosis after SCI

**Fig. 1:**
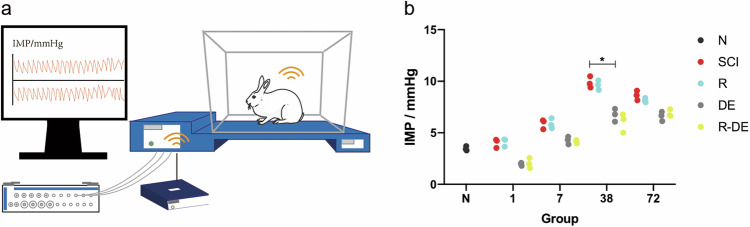
IMP significantly decreased after surgical decompression. **a** Illustration of IMP measurement. IMP was measured using a Millar Telemetry System. **b** IMP increased after SCI, and IMP significantly decreased after surgical decompression. Individual data points are presented, *n* = 3. Two-way repeated measures ANOVA followed by Sidak’s multiple comparisons test. *P < 0.05.

To observe the effects of surgical decompression and RAPA on IMP after SCI, we monitored the IMP in the normal (N), SCI, rapamycin (R), surgical decompression (DE), and rapamycin combined with surgical decompression (R-DE) treatment groups (Fig. 1a). Compared with the N group, the SCI group exhibited increased IMP, peaking at 38 h, at which time the DE group showed significantly decreased IMP (*p* = 0.0342) and the R-DE group showed a decreasing trend, although this change was not significant (*p* = 0.0706). These results showed that IMP decreased after decompression. Compared with the SCI group, there was no significant change in IMP after the RAPA intervention. There were no significant changes in the R-DE group compared to DE group, indicating that RAPA may not have a significant impact on IMP (Fig. 1b).

**Fig. 2:**
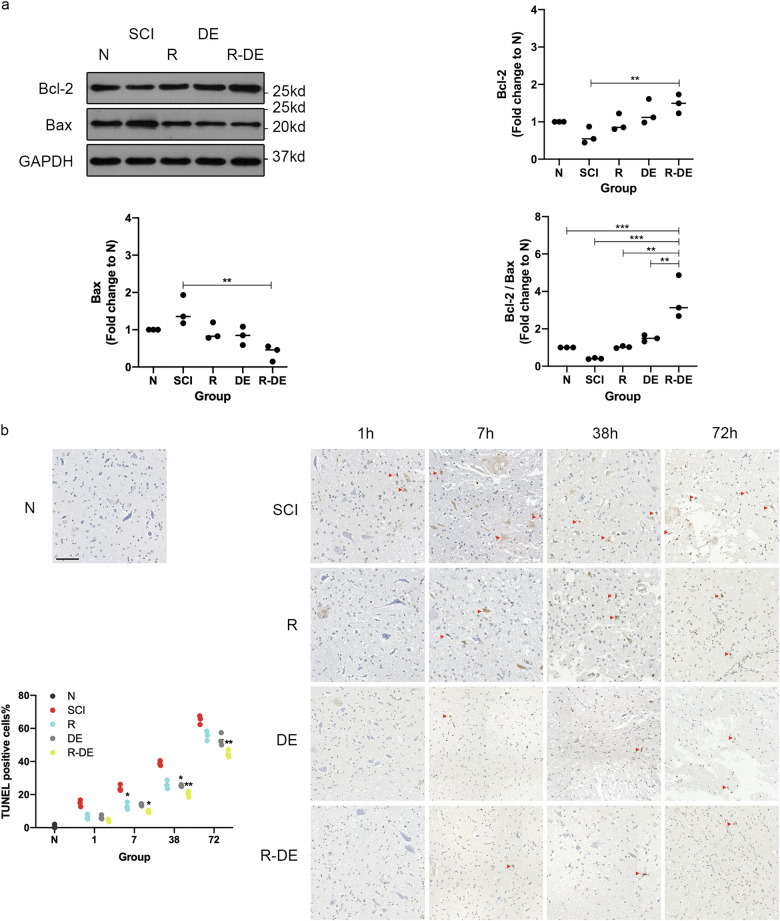
Combination therapy significantly ameliorated apoptosis after SCI. **a** WB analysis of the levels of Bcl-2 and Bax proteins in spinal cord lysates obtained from the N, SCI, R, DE, and R-DE groups 72 h after injury. GAPDH was used as the loading control. Bcl-2 and Bax protein levels and the Bcl-2/Bax ratio were quantified, and the fold change relative to N was calculated. Individual data points and medians are presented, *n* = 3. One-way ANOVA followed by Tukey’s multiple comparisons test. **P < 0.01, ***P < 0.001. **b** Images of apoptotic cells in spinal cord sections obtained from the N, SCI, R, DE, and R-DE groups assessed using TUNEL staining 1 h, 7 h, 38 h, and 72 h after injury. The red arrows indicate TUNEL-positive cells. Scale bar = 100 μm. The TUNEL-positive cells% in the spinal cord sections was quantified. Individual data points are presented, *n* = 3. Two-way repeated measures ANOVA followed by Sidak’s multiple comparisons test. *P < 0.05 and **P < 0.01 compared to the SCI group.

To investigate the effect of surgical decompression and RAPA on apoptosis after SCI, we examined the protein expression of Bax and Bcl-2 in the 72 h postinjury in the N, SCI, R, DE, and R-DE groups. Compared with the N group, Bcl-2 was decreased, Bax was increased and the Bcl-2/Bax ratio was decreased in the SCI group, indicating that apoptosis was promoted after injury. Compared with the SCI group, the R-DE group exhibited significantly higher Bcl-2 (*p* = 0.0078), lower Bax (*p* = 0.0022) expression levels, and a higher Bcl-2/Bax ratio (*p* = 0.0002), indicating that apoptosis was inhibited in the R-DE group (Fig. 2a). Compared with the N group, the number of TUNEL-positive cells was significantly higher in the SCI group, indicating that apoptosis increased after SCI. Compared with the SCI group, the number of positive cells was slightly decreased in the R and DE groups and significantly decreased in the R-DE group at 7 h, 38 h, and 72 h (*p* = 0.0364, 0.0018, 0.0069 at 7 h, 38 h, and 72 h, respectively). In addition, the R-DE group showed a decreasing trend at 1 h, but no significant difference was observed (p = 0.0721) (Fig. 2b). Taken together, these data suggest that surgical decompression combined with RAPA significantly inhibited apoptosis after SCI.

### RAPA combined with surgical decompression significantly improved autophagic flux after SCI

**Fig. 3:**
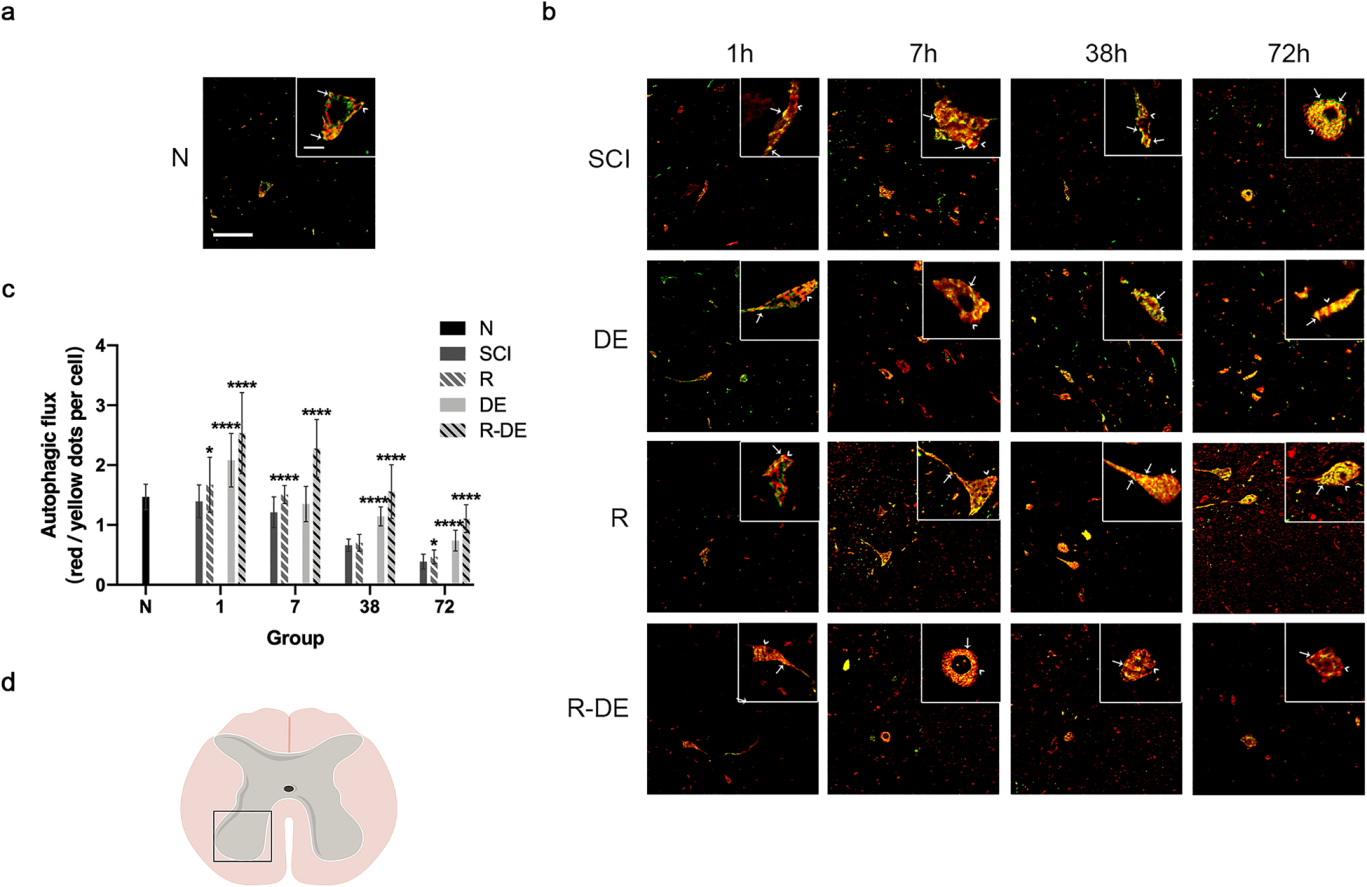
Autophagic flux was impaired after SCI and significantly relieved after combination therapy. **a** Images of spinal cord sections obtained from rabbits in the N group injected with AAV-mRFP-GFP-LC3. **b** Images of spinal cord sections obtained from rabbits in the SCI, R, DE, and R-DE groups injected with AAV-mRFP-GFP-LC3. The arrowheads indicate red dots. The arrows indicate yellow dots. Scale bar = 100 μm (10×). Scale bar = 10 μm (40×). **c** The autophagic flux (red/yellow dot ratio per cell) was quantified. The data are presented as the means ± SDs, *n* = 3. More than 10 neurons were quantified for each slide. Two-way repeated measures ANOVA followed by Sidak’s multiple comparisons test. *P < 0.05 and ****P < 0.0001 compared to the SCI group. **d** The schematic drawing illustrates the selected regions of interest for autophagic flux quantification.

To observe the effect of surgical decompression and RAPA on autophagic flux after SCI, we measured the autophagic flux (red/yellow dot ratio) in the N, SCI, R, DE, and R-DE groups. The results showed that compared with the N group, the SCI group exhibited a decreased red/yellow dot ratio, indicating that the autophagic flux was impaired after SCI. Compared with the SCI group, the R, DE, and R-DE groups showed increased autophagic flux, with the most significant increase in the R-DE group (*p* < 0.0001 at 1 h, 7 h, 38 h, and 72 h) (Fig. 3a–d). Taken together, these data suggest that surgical decompression combined with RAPA significantly improved autophagic flux after SCI.

### RAPA combined with surgical decompression significantly improved the number of neurons and inhibited demyelination after SCI

**Fig. 4:**
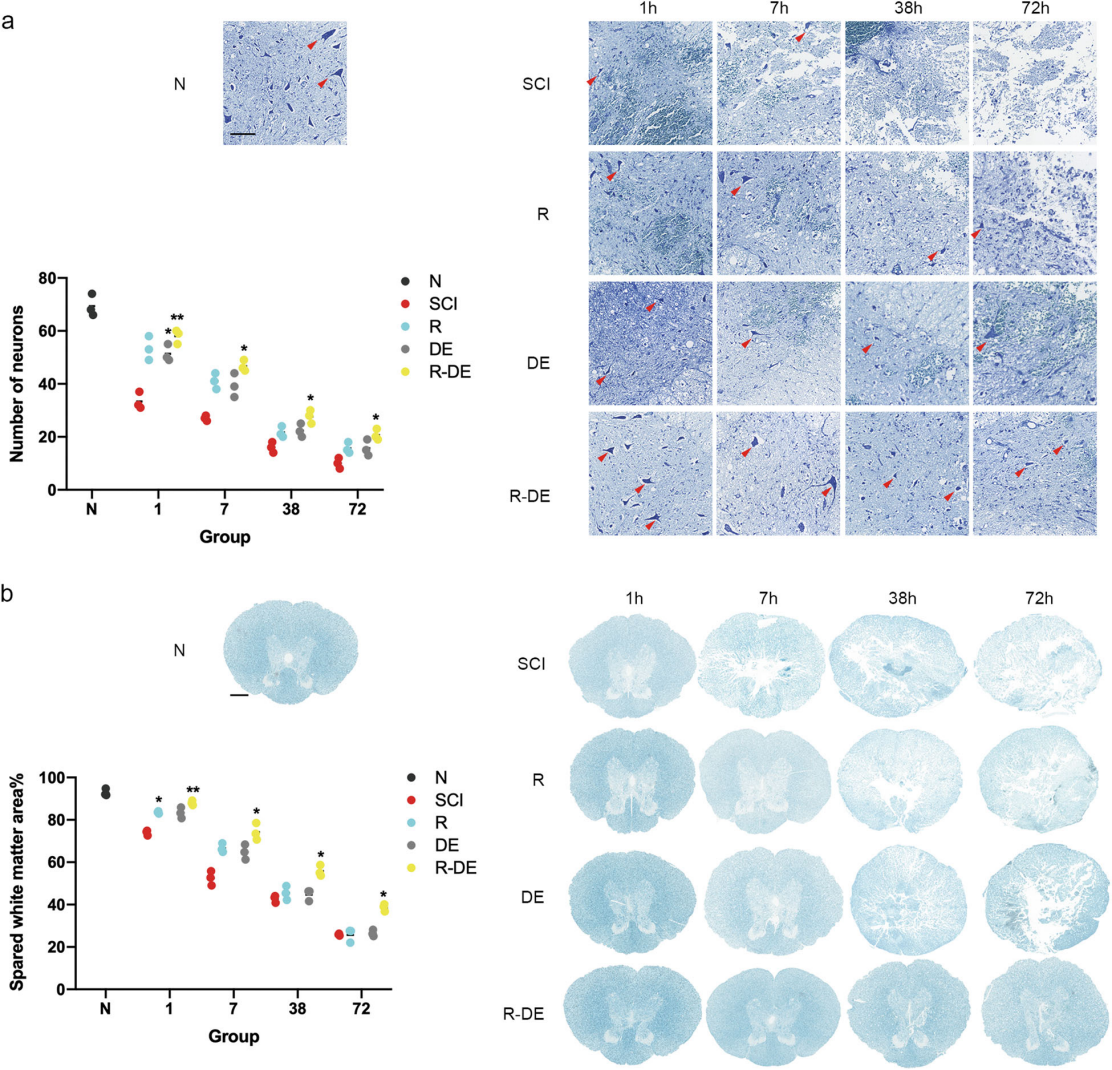
Combination therapy significantly improved the neurons and inhibited demyelination after SCI. **a** Images of neurons in spinal cord sections obtained from the N, SCI, R, DE, and R-DE groups assessed using Nissl staining 1 h, 7 h, 38 h, and 72 h after injury. The arrows indicate neurons. Scale bar = 100 μm. The numbers of neurons in the spinal cord sections were quantified. Individual data points are presented, *n* = 3. Two-way repeated measures ANOVA followed by Sidak’s multiple comparisons test. *P < 0.05 and **P < 0.01 compared to the SCI group. **b** Images of spinal cord sections obtained from the N, SCI, R, DE, and R-DE groups assessed using LFB staining 1 h, 7 h, 38 h, and 72 h after injury. Scale bar = 500 μm. The spared white matter area in the spinal cord sections was quantified. Individual data points are presented, *n* = 3. Two-way repeated measures ANOVA followed by Sidak’s multiple comparisons test. *P < 0.05 and **P < 0.01 compared to the SCI group.

**Fig. 5:**
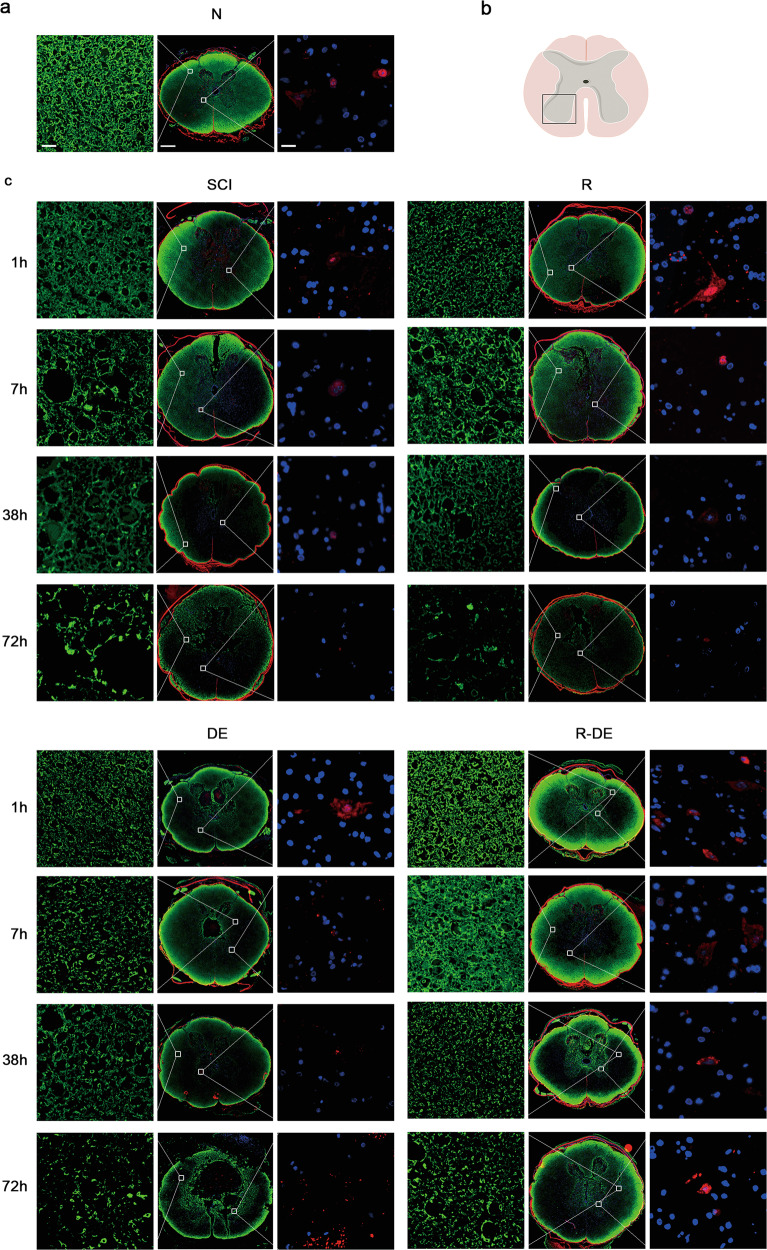

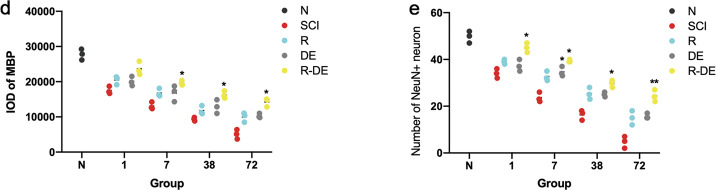
Spinal cord neurons and myelin sheaths were significantly improved after combination therapy. **a** Images of sections of injured spinal cords from rabbits in the N group. The sections were stained with antibodies against MBP and NeuN. Scale bars = 20 μm, 500 μm, and 20 μm (from left to right). **b** The schematic drawing illustrates the selected regions of interest for quantification of the number of NeuN^+^ neurons. **c** Images of sections of injured spinal cords from rabbits in the SCI, R, DE, and R-DE groups 1 h, 7 h, 38 h, and 72 h after injury. **d** The IOD of MBP staining in the spinal cord sections was quantified. Individual data points are presented, *n* = 3. Two-way repeated measures ANOVA followed by Sidak’s multiple comparisons test. *P < 0.05 compared to the SCI group. **e** The number of NeuN^+^ neurons in the spinal cord sections was quantified. Individual data points are presented, *n* = 3. Two-way repeated measures ANOVA followed by Sidak’s multiple comparisons test. *P < 0.05 and **P < 0.01 compared to the SCI group.

To investigate the effect of surgical decompression and RAPA on neurons and myelin sheaths after SCI, we performed Nissl and Luxol fast blue (LFB) staining in the N, SCI, R, DE, and R-DE groups and quantified the neurons in the anterior horn of the spinal cord and residual white matter area. The results showed that the number of neurons in the anterior horns of the spinal cord and the area of residual white matter were significantly lower in the SCI group than in the N group. In addition, significantly higher numbers of neurons were observed in the R-DE group than in the SCI group, indicating that the combination therapy significantly improved the number of neurons after SCI (Fig. 4a, b). We also performed immunofluorescence staining for MBP and NeuN (Fig. 5a–c). Compared with the N group, the SCI group showed a significantly decreased integrated optical density (IOD) of MBP staining and a significantly decreased number of NeuN^+^ cells. Compared with the SCI group, the three intervention groups showed an increased IOD of MBP staining, with the highest value observed in the R-DE group (*p* = 0.1657, 0.0179, 0.0277, 0.0114 at 1 h, 7 h, 38 h, and 72 h, respectively), which suggests that the combination therapy inhibited demyelination after SCI (Fig. 5d). Compared with the SCI group, the number of NeuN^+^ neurons was increased in the three intervention groups, with the best effect observed in the R-DE group (*p* = 0.0300, 0.0421, 0.0152, 0.0080 at 1 h, 7 h, 38 h, and 72 h, respectively), indicating that the combination therapy significantly improved the number of NeuN^+^ neurons (Fig. 5e).

### Reduced IMP promotes autophagy

**Fig. 6:**
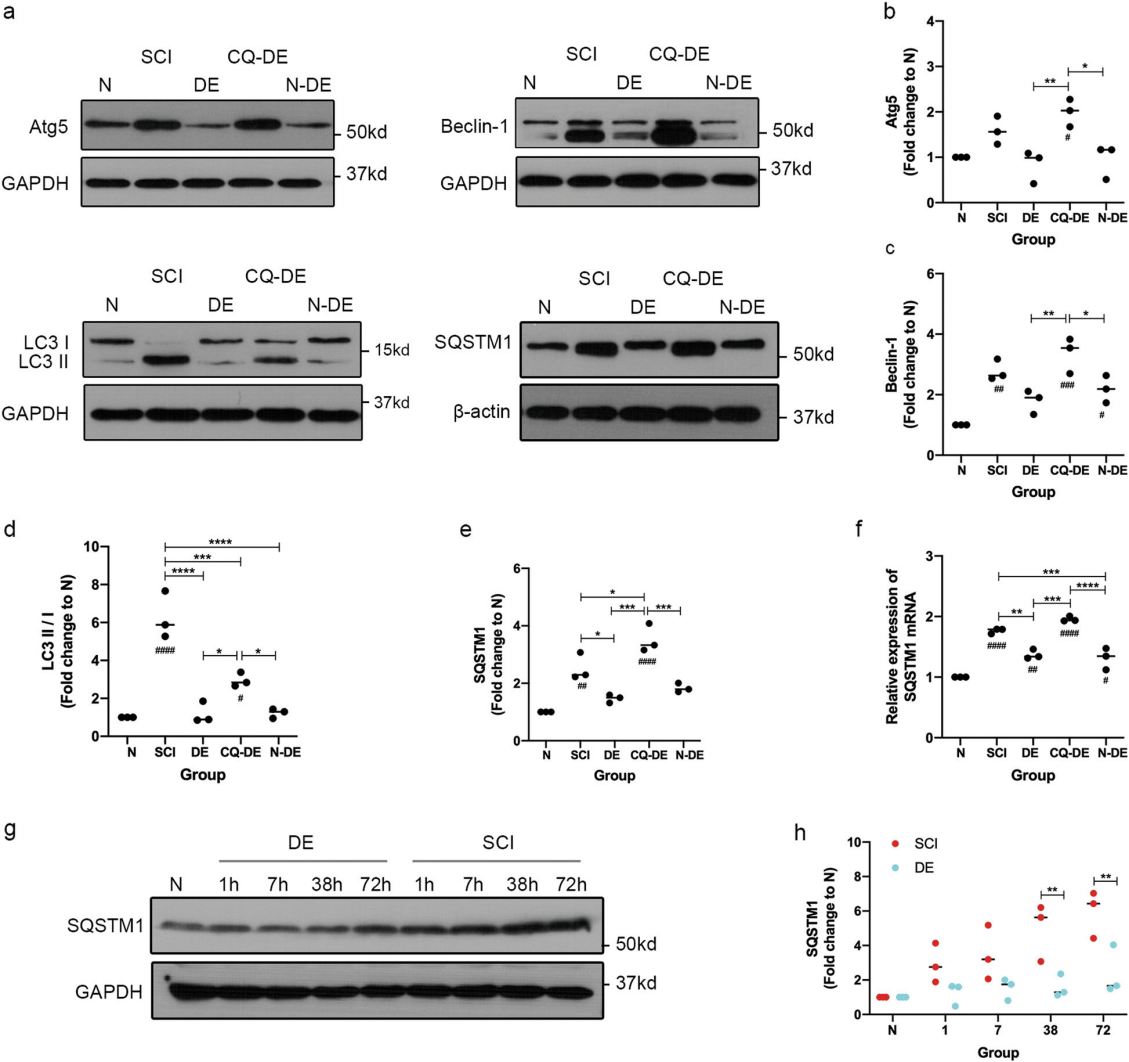
Altered autophagy-related protein expression after SCI and decompression. **a** WB analysis of the levels of the autophagy-related proteins LC3, Beclin-1, Atg5, and SQSTM1 in spinal cord lysates obtained from the N, SCI, DE, CQ-DE, and N-DE groups. β-actin or GAPDH was used as the loading control. **b**–**e** Atg5, Beclin-1, LC3 II/I, and SQSTM1 protein levels were quantified, and fold change to N was calculated. Individual data points and medians are presented, *n* = 3. One-way ANOVA followed by Tukey’s multiple comparisons test. *P < 0.05, **P < 0.01, and ***P < 0.001; ^#^*P* < 0.05, ^##^*P* < 0.01, ^###^*P* < 0.001, and ^####^*P* < 0.0001 compared to the N group. **f** qPCR of the levels of SQSTM1 mRNA in spinal cord lysates obtained from the N, SCI, DE, CQ-DE, and N-DE groups. Individual data points and medians are presented, *n* = 3. One-way ANOVA followed by Tukey’s multiple comparisons test. **P < 0.01, ***P < 0.001, and ****P < 0.0001; ^#^*P* < 0.05, ^##^*P* < 0.01, and ^####^*P* < 0.0001 compared to the N group. **g** WB analysis of the levels of SQSTM1 protein in spinal cord lysates obtained from the N, SCI, and DE groups 1 h, 7 h, 38 h, and 72 h after injury. GAPDH was used as the loading control. **h** SQSTM1 protein levels were quantified, and the fold change relative to N was calculated. Individual data points and medians are presented, *n* = 3. Two-way repeated measures ANOVA followed by Sidak’s multiple comparisons test. **P < 0.01.

To further evaluate the effect of IMP on autophagy after SCI, we assessed Beclin-1, LC3, Atg5, and SQSTM1 protein expression. Compared with the N group, the SCI group exhibited increased SQSTM1 protein expression, indicating that increased IMP may impair autophagic degradation. In addition, increased levels of Beclin-1, LC3 II/I, and Atg5 protein expression were observed, indicating an accumulation of autophagy initiation-related proteins. Compared with the SCI group, the DE group exhibited significantly lower SQSTM1 protein expression, indicating that autophagic degradation was promoted. In addition, significantly lower Beclin-1, LC3 II/I, and Atg5 protein expression was observed, suggesting the autophagy initiation-related proteins that accumulated due to damage were consumed. However, the levels of Beclin-1, LC3 II/I, Atg5, and SQSTM1 proteins were higher in the chloroquine (CQ)-DE group compared with that observed in the control (N-DE) group, indicating that autophagy was inhibited by CQ. Compared with the DE group, no significant differences in these proteins were observed in the N-DE group, indicating that the vehicle solvent did not exert any effects (Fig. 6a–e). Compared with the SCI group, the DE group exhibited lower SQSTM1 protein expression at 1 h, 7 h, 38 h, and 72 h, indicating that decompression promotes autophagic degradation (Fig. 6g, h). Because an incorrect interpretation of autophagy could occur when only the SQSTM1 protein level is measured, we measured the SQSTM1 mRNA levels. Compared with the N group, the SCI group exhibited increased SQSTM1 mRNA levels (*p* < 0.0001), indicating that increased transcription of SQSTM1 may be related to the increased IMP after SCI. Compared with the SCI group, the DE group exhibited significantly lower SQSTM1 mRNA levels (*p* = 0.0025), suggesting that decreased transcription of SQSTM1 may be related to the decreased IMP. The SQSTM1 mRNA levels were significantly higher in the CQ-DE group than those observed in the N-DE group (*p* < 0.0001), indicating that CQ may affect SQSTM1 transcription after decompression (Fig. 6f).

## Discussion

This study investigated the therapeutic effects of RAPA and decompression, in combination or separately, on the histopathology of the injured spinal cord in a rabbit model of SCI. The combination therapy achieved a better effect compared with RAPA/surgical decompression alone. Furthermore, our data indicate that increased IMP after SCI inhibited autophagic degradation and impaired autophagic flux. Decompression improved autophagic flux after SCI.

Tissue hemorrhage, edema, the inflammatory response, and other factors after SCI cause increased IMP, thereby exacerbating secondary injury and affecting neurological function^24–28^. In this study, IMP was significantly reduced after dural incision in a rabbit model of SCI. Although RAPA inhibits inflammatory cell infiltration and proinflammatory factor production and improves the microenvironment after SCI^17,29^, this study shows that compared with decompression alone, combination therapy within 72 h after SCI led to no significant difference in IMP. Longer monitoring may yield different results. In this study, after RAPA/surgical decompression alone, Bcl-2 expression increased slightly and Bax expression decreased slightly. In the combination group, Bcl-2 expression was significantly increased, while Bax expression was significantly reduced. Increased Bcl-2 and decreased Bax expression indicate increased resistance to apoptosis^30^. The number of TUNEL-positive cells decreased and the number of residual neurons in the anterior horns of the spinal cord increased after combination therapy more than after the other therapies. These results indicate that combination therapy after SCI significantly inhibits apoptosis and protects residual neurons. The residual white matter area and MBP expression in the combination group were significantly higher than those in the other groups, indicating that the combination therapy significantly inhibits demyelination. Autophagy is a dynamic process that includes autophagosome assembly, the delivery of autophagic substrates to lysosomes, and autolysosomal degradation. The entire process is usually described as “autophagic flux”^31^. In this study, autophagic flux decreased after SCI. Compared with SCI, RAPA treatment alone induced autophagy and alleviated autophagic flux obstruction caused by injury. These results are consistent with the results of a study by Castillo et al.^31^. At the same time point after injury, autophagic flux was significantly higher in the combination therapy group than in the other groups. These results indicate that combination therapy significantly enhanced autophagic flux. Autophagic flux also increased after decompression alone, indicating that IMP may has some effects on autophagy after SCI.

The effects of IMP on autophagy after SCI was explored in this study. The conversion of LC3 I to LC3 II is considered to be a marker of autophagic activity^32^. Atg5 is involved in autophagy induction and autophagosome formation and is an essential protein for autophagic flux^33^. The Beclin-1 complex is involved in autophagosome formation during the initiation of autophagy^34,35^. The expression of these three autophagy initiation-related proteins increased after SCI in this study, which is consistent with the results of previous studies^12,36–38^. The level of SQSTM1 is an indicator of autophagic degradation^39,40^. We found that SQSTM1 expression increased and autophagic flux decreased after SCI, which is consistent with the results of previous studies^41,42^. Considering that autophagy initiation-related protein levels increased, whereas autophagic flux decreased after SCI, autophagic degradation could be impaired. This means that the increased IMP may impair autophagic degradation. However, the mRNA level of SQSTM1 also increased after SCI. It is likely that the elevation of SQSTM1 levels was also partly caused by the induction of its transcription. LC3 transport blockade caused by injury prevents accumulated autophagosomes from being effectively degraded^31^. After decompression, the expression of all three autophagy initiation-related proteins decreased, whereas autophagic flux increased. This may have occurred because decompression promotes autophagic degradation, thereby consuming the autophagosomes that accumulated due to damage. The decrease in SQSTM1 protein expression may be affected by the simultaneous enhancement of autophagic degradation and the reduction of transcription. Compared with the N-DE group, the expression of all three autophagy initiation-related proteins was increased in the CQ-DE group, indicating that lysosomes and proteasomes were inhibited by CQ and the accumulation of autophagosomes. Compared with the N-DE group, the accumulation of SQSTM1 protein in the CQ-DE group may be affected by the simultaneous decrease in autophagic degradation and increase in protein synthesis. In this study, changes in SQSTM1 mRNA and protein levels occurred in the same direction in groups. Decompression reversed the increased SQSTM1 mRNA levels caused by SCI, indicating that transcription of SQSTM1 may be affected by IMP. CQ may affect SQSTM1 transcription after decompression, and the mechanism requires further study. In this study, considering that SQSTM1 participates in a variety of processes^43–45^, changes in SQSTM1 protein levels after decompression may not only represent changes in autophagic degradation, but may also be involved in other processes (such as mechanical signaling) to affect pathological changes of the spinal cord after SCI, which requires intensive study in the future.

The results of this study showed that RAPA treatment combined with surgical decompression therapy had better histopathological effects in rabbits with SCI compared with RAPA/surgical decompression alone, and it is expected to become a new treatment for SCI. This study did have some limitations. For example, IMP measurements would be more accurate in large animals (such as rabbits), but the sample size would be smaller. This combination therapy should be further studied to evaluate its therapeutic potential as a method for treating SCI. In addition, the effects of IMP on autophagy after SCI was explored in this study for the first time. Studies have reported that autophagic flux increases with injury severity and plays different roles in mild and severe injury^18^. Early autophagy induction in rats with spinal cord ischemia/reperfusion injury inhibited apoptosis and inflammation, while autophagy induction led to cell death and aggravated injury 72 h after the initial injury^46^. Part of the reason for these phenomena may be the different degrees of injury associated with a variety of IMPs, but further research is needed to evaluate this possibility.

In summary, the results of this study revealed that RAPA combined with surgical decompression therapy has a better effect on the histopathology of rabbits with SCI compared with RAPA/surgical decompression alone. Increased IMP after SCI inhibited autophagic degradation and impaired autophagic flux. Decompression improved autophagic flux after SCI.

## Materials and methods

### Animals

Healthy female Japanese white rabbits weighing 2.0 ± 2.5 kg and aged 2.5 ± 3.0 months were used in the study (Beijing Jinmuyang Experimental Animal Breeding Co., Ltd., Beijing, China). All animals were allowed free access to food and water. All experimental procedures were approved by the local ethics committee and were in accordance with the guidelines for animal use and care. The study was conducted according to the ethical rules of the Animal Experiments and Experimental Animal Welfare Committee (AEEI-2018-008).

### Reagent preparation and injection

RAPA was used to induce autophagy. DMSO (D4540, Sigma-Aldrich), PEG (202371, Sigma-Aldrich), and Tween 80 (P1754, Sigma-Aldrich) were added to RAPA (S1039, Selleck Chemicals, US) (2% DMSO + 30% PEG 300 + 5% Tween 80 + ddH_2_O). Two hours before surgery, RAPA (2 mg/kg) was injected into the marginal ear vein of rabbits in the R and R-DE groups. To maintain efficacy, the same dose of drug was injected again 36 h after surgery. CQ was given as an autophagy-lysosomal pathway inhibitor to inhibit autophagy after surgery in the CQ-DE group. CQ (c6628, Sigma-Aldrich, St. Louis, MO, USA) was dissolved in 150 mg/ml physiological saline and administered intraperitoneally at 60 mg/kg/d^47^. The N-DE group was injected with the same volume of vehicle solvent.

### Surgery

The animals were fasted for 12 h before surgery, and sodium pentobarbital (3%, 30 mg/kg) was injected into the ear vein for anesthesia. In each rabbit, a spinal contusion at the T10 level was created using an aneurysm clip (REBSTOCK, Dürbheim, Tuttlingen, Germany) at an intensity of 90 g and with a retention time of 1 min. Tail-spasm swings and strong lower limb contractions were used as indicators for evaluating successful establishment of the SCI rabbit model. Animals in the N group underwent laminotomy only. Dural incisions were made in the DE group immediately after injury. The dura mater was cut with a number 11 scalpel and rinsed with saline. The length of the incision was ~2 mm. The cavity created by the removed spine was filled with a gelatin sponge, and the muscles, fascia, and skin were sutured. Sodium Lactate Ringer’s Injection (Shandong Qidu Pharmaceutical Co., Ltd., China) was injected into the marginal vein during the operation until the animal fully recovered. Subcutaneous injections of penicillin sodium (North China Pharmaceutical Co., Ltd., China) and gabapentin (Hainan Selection Pharmaceutical Co., Ltd., China) were administered intragastrically for 3 days postoperatively, and urination was artificially assisted 3–4 times a day. All surgeries are performed by one surgeon. After SCI, all rabbits were assigned to a treatment group according to a randomized block experimental design. The number of rabbits at various time points in each study is indicated in the figure legends.

### IMP monitoring

IMP was measured using a Millar Telemetry System (MKT0002/D, Millar, Houston, TX), as previously described^48^. IMP was recorded at 1 h, 7 h, 38 h, and 72 h after SCI in the N, SCI, R, DE, and R-DE groups (*n* = 3 per group). All animals were continuously monitored for 1 h at the corresponding time points, and the results were averaged.

### Autophagic flux measurement

Two weeks before SCI modeling, animals were intrathecally injected with AAV-mRFP-GFP-LC3 (purchased from Hanbio, Shanghai, China) for subsequent autophagic flux measurement. Using a stereotactic device (51750, Stoelting, Wood Dale, USA) and a microinjection pump (53311, Stoelting) equipped with a 25 μl microinjector (Hamilton, Reno, NV, USA) and a 33 G needle, the animals were internally injected with 20 µl of construct at a speed of 1 μl/min. After the injection was completed, the injection needle was left in place for 20 min., and the wound was sutured after the needle was pulled out. At the corresponding time point, the spinal cord was removed by the above method and then dehydrated and embedded in paraffin. For each group, *n* = 3, and each sample was cut into coronal sections (5 μm) at the center of the injury. After the images were scanned with a Pannoramic 250 system, the two channels were merged for colocalization analysis (i.e., red and yellow dot analysis), and the red and yellow dots were manually counted in a double-blind manner. GFP, but not mRFP, degrades in an acidic environment^49^. Therefore, yellow dots (merged red and green dots) represent autophagosomes, while red dots represent autophagolysosomes. If autophagy is activated and autophagic flux is normal, red dots will dominate over yellow dots; if autophagic flux is impaired, more yellow dots than red dots will be observed^50,51^.

### Statistical analysis

GraphPad Prism 8.0 (GraphPad Software, San Diego, California, USA) was used in this study for statistical analysis. The number of animals in all studies was determined by power analysis (power of 0.8 with alpha value of 0.05). Key experiments were repeated with independent groups of animals to ensure reproducibility. Two-way repeated measures ANOVA followed by Sidak’s multiple comparisons test was used to analyze differences among the SCI, R, DE, and R-DE groups. One-way ANOVA followed by Tukey’s multiple comparisons test was used to analyze differences among the N, SCI, DE, CQ-DE, and N-DE groups. *P* < 0.05 was considered to indicate statistical significance.

## Supplementary information

Supplementary Table

Supplementary information
