# Correction: Therapeutic effects of rapamycin and surgical decompression in a rabbit spinal cord injury model

**DOI:** 10.1038/s41419-020-02829-8

**Published:** 2020-08-10

**Authors:** Xin Zhang, Chuan Qin, Yingli Jing, Degang Yang, Changbin Liu, Feng Gao, Chao Zhang, Zuliyaer Talifu, Mingliang Yang, Liangjie Du, Jianjun Li

**Affiliations:** 1 School of Rehabilitation Medicine, Capital Medical University, Beijing, 100068 China; 2 China Rehabilitation Science Institute, Beijing, 100068 China; 3 Center of Neural Injury and Repair, Beijing Institute for Brain Disorders, Beijing, 100068 China; 4 Department of Spinal and Neural Functional Reconstruction, China Rehabilitation Research Center, Beijing, 100068 China; 5 Beijing Key Laboratory of Neural Injury and Rehabilitation, Beijing, 100068 China; 6 Institute of Rehabilitation Medicine, China Rehabilitation Research Center, Beijing, 100068 China; 7 Department of Rehabilitation Medicine, Beijing Tiantan Hospital, Beijing, 100050 China

Correction to: *Cell Death & Disease*

10.1038/s41419-020-02767-5, published online date 23 July 2020

In the original version of this article, Fig. 6 appeared incorrectly in the PDF. It showed panels d and e of Fig. 5 instead of the intended Fig. 6, which was omitted. This has now been corrected in the PDF version of the Article.

