# Correction: Therapeutic effects of rapamycin and surgical decompression in a rabbit spinal cord injury model

**DOI:** 10.1038/s41419-025-08267-8

**Published:** 2025-12-23

**Authors:** Xin Zhang, Chuan Qin, Yingli Jing, Degang Yang, Changbin Liu, Feng Gao, Chao Zhang, Zuliyaer Talifu, Mingliang Yang, Liangjie Du, Jianjun Li

**Affiliations:** 1 School of Rehabilitation Medicine, Capital Medical University, 100068 Beijing, China; 2 China Rehabilitation Science Institute, 100068 Beijing, China; 3 Center of Neural Injury and Repair, Beijing Institute for Brain Disorders, 100068 Beijing, China; 4 Department of Spinal and Neural Functional Reconstruction, China Rehabilitation Research Center, Beijing, 100068 China; 5 Beijing Key Laboratory of Neural Injury and Rehabilitation, 100068 Beijing, China; 6 Institute of Rehabilitation Medicine, China Rehabilitation Research Center, 100068 Beijing, China; 7 Department of Rehabilitation Medicine, Beijing Tiantan Hospital, 100050 Beijing, China

Correction to: *Cell Death and Disease* 10.1038/s41419-020-02767-5 published online 23 July 2020

The original version of this article contained an error where an incorrect version of Fig. 3 was published. The figure should have appeared as shown below.

**Original data Fig 3** 
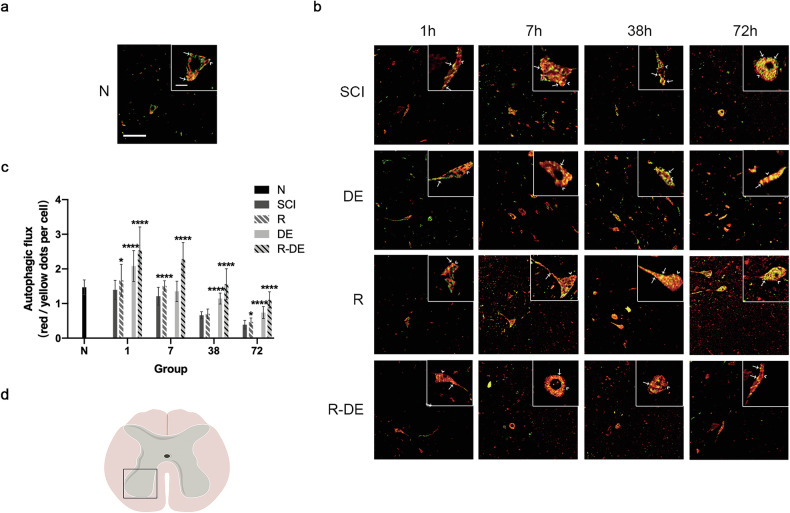


**Amended file-Fig 3** 
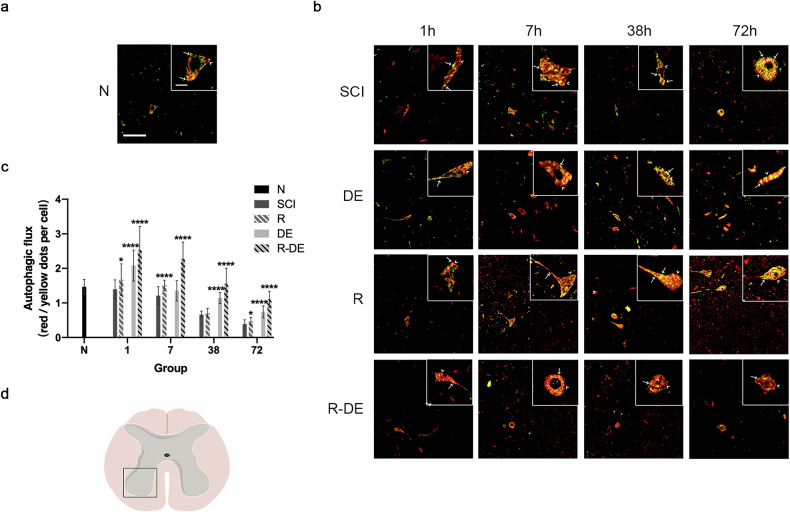


The original article has been corrected.

