# Supplementary material for: Therapeutic effects of rapamycin and surgical decompression in a rabbit spinal cord injury model
Source: Cell Death Dis. 2020 Jul 23;11(7):567. doi: 10.1038/s41419-020-02767-5 (PMC7378229; doi:10.1038/s41419-020-02767-5)
Supplement: Supplementary file 1 — Supplementary Table [file 41419_2020_2767_MOESM1_ESM.docx]

**Supplementary Table 1.** Antibodies information

| Antibodies | Source | Identifier | Application |
| --- | --- | --- | --- |
| Anti-NeuN | Millipore, Bedford, MA, USA | MAB377 | IF |
| Anti-Myelin Basic | Abcam, Cambridge, UK | ab62631 | IF |
| Goat Anti-Mouse (HRP) | Servicebio, Wuhan, China | GB23301 | IF |
| Goat Anti-Mouse (488) | Servicebio, Wuhan, China | GB25301 | IF |
| β-actin | Abcam, Cambridge, UK | ab8226 | WB |
| GAPDH | Abcam, Cambridge, UK | ab8245 | WB |
| LC3B | Cell Signaling Technology, Danvers, MA, USA | 2775S | WB |
| Atg5 | Cell Signaling Technology, Danvers, MA, USA | 12994S | WB |
| SQSTM1/p62 | LifeSpan Biosciences, Seattle, WA, USA | LS-C313090 | WB |
| Beclin-1 | Cell Signaling Technology, Danvers, MA, USA | 3738S | WB |
| Bcl-2 | Proteintech, Wuhan, China | 12789-1-AP | WB |
| Bax | Proteintech, Wuhan, China | 50599-2-Ig | WB |
| Anti-mouse IgG (HRP) | Cell Signaling Technology, Danvers, MA, USA | 7076S | WB |
| Anti-rabbit IgG (HRP) | Cell Signaling Technology, Danvers, MA, USA | 7074S | WB |
