# Supplementary material for: Therapeutic effects of rapamycin and surgical decompression in a rabbit spinal cord injury model
Source: Cell Death Dis. 2020 Jul 23;11(7):567. doi: 10.1038/s41419-020-02767-5 (PMC7378229; doi:10.1038/s41419-020-02767-5)
Supplement: Supplementary file 2 — Supplementary information [file 41419_2020_2767_MOESM2_ESM.docx]

**Supplemental Material**

**MATERIALS AND METHODS**

***Histology, image acquisition, and quantification***

The spinal cord was removed immediately after each animal was sacrificed by anesthesia at the corresponding time point. A spinal cord segment with a length of 5 mm was taken from around the point of injury (T10) and fixed in 4% paraformaldehyde for 24 hours. Then, dehydration and paraffin embedding were performed, followed by cross-sectioning at a thickness of 4 μm. After Nissl and LFB staining, images were captured at a high resolution with HistoFAXS 3.0 (TissueGnostics, Vienna, Austria), and the images were viewed using the associated professional viewing software (FAXS viewer). All images were acquired 2–3 mm rostral or caudal to the epicenter, and 8 to 10 sections from each sample were imaged. Images were captured from n = 3 rabbits per group. For each experiment, data from all images for each rabbit were averaged and used for final statistical analysis. The Nissl-positive cells in the anterior horns of the spinal cord were manually counted (positive definition: intact cells, nuclei and axons) in a double-blind manner, and the counts were averaged. For quantification, cross-sectional images were captured at 20× magnification and digitized using Image-Pro Plus 6.0 (Media Cybernetics, Silver Spring, MD). The threshold level of each 8-bit image was set to label only LFB-positive tissue and calculate the total LFB-positive area (residual white matter area) for each section. The percentage of residual white matter area was calculated as follows: residual white matter area = residual white matter area/(total area-gray matter area) %. All quantization was performed using original, unedited images.

***Immunofluorescence, image acquisition, and quantification***

After paraffin sections were dewaxed, they were placed in citric acid antigen retrieval buffer (pH = 6.0) in a microwave oven for antigen retrieval. After the sections were blocked with serum, they were incubated with primary antibodies (see Supplementary Table 1 for reagent use) at 4°C overnight and then secondary antibodies, and then nuclei were counterstained with DAPI. The fluorescence images were captured with a Pannoramic 250 system (Pannoramic MIDI; 3D HISTECH, Budapest, Hungary), and then the images were viewed with CaseViewer (3D HISTECH) software. NeuN-positive cells in the anterior horns of the spinal cord were manually counted in a double-blind manner, and the counts were averaged. MBP immunofluorescence staining was used to observe the myelin sheath conditions. For quantification, the cross-sectional images were captured at 2x magnification in CaseViewer and digitized using Image-Pro Plus. After background correction, the IOD of MBP staining from the section as a whole was automatically detected in a double-blind manner. Statistical analysis was then performed. All quantization was performed using original, unedited images. All images were processed with Photoshop CC (Adobe, San Jose, California, USA) software. All images were acquired 2–3 mm rostral or caudal to the epicenter, and 10 sections per sample were imaged. Images were captured from n = 3 rabbits per group. For each experiment, data from all images for each rabbit were averaged and used for final statistical analysis.

***TUNEL***

TUNEL was used to detect apoptosis in anterior horn neurons of the spinal cord. To block endogenous peroxidase activity, sections were incubated with a methanol solution containing 0.2% H_2_O_2_ for 0.5 hours. Then, the sections were treated with a TUNEL reaction mixture (Millipore, Bedford, MA, USA), and the sections were kept in a 37°C incubator for 60 minutes. After HistoFAXS 3.0 was used to scan all tissue section images, the total numbers of cells in the anterior horns of the spinal cord and the numbers of TUNEL-positive cells were manually counted in a double-blind manner. The ratio was calculated as follows: TUNEL-positive cells% = (TUNEL-positive cells)/(total cells) × 100%^1^.

***Western blot (WB) analysis***

The spinal cord samples were frozen in liquid nitrogen immediately after removal and stored at -80°C. Tissue homogenate protein extracts were used for WB detection (n = 3 per group), and the expression of several proteins was analyzed (Supplementary Table 1). Protein from the spinal cord was separated by 10% SDS-PAGE and transferred to a PVDF membrane. The membrane was incubated with primary antibodies at 4°C overnight. The membrane was then incubated with secondary antibodies under shaking, developed in a dark room, and scanned after fixation. The band gray values were quantified by Image-Pro Plus and are expressed as ratios relative to the values for β-actin or GAPDH.

***RNA isolation and qPCR***

Total RNA was extracted from the spinal cord or from primary cell cultures using RNA extraction reagent (G3013, Servicebio, Wuhan, China). First-strand cDNA was synthesized using a RevertAid First-Strand cDNA Synthesis Kit (#K1622, Thermo Scientific Fermentas, Vilnius, Lithuania). qPCR was performed on a Real Time PCR system (StepOne Plus, Applied Biosystems ABI, CA, USA). Gene expression was expressed as the mRNA level, which was normalized to the mRNA level of a standard housekeeping gene (β-actin) using the ΔΔCT method. At least three independent experiments were performed for each set of PCR analyses. The primers used for qPCR were as follows: SQSTM1, F (5’-AACCCGCTACAAGTGCAGTGTC-3’) and R (5’-CGAAGTGCCCATGTCTCAGC-3’); and β-actin, F (5’-CGCAGAAACGAGACGAGATTG-3’) and R (5’-GATGCTCGCTCCAACGACTG-3’).

**REFERENCES**

1. Shao, L. et al. SENP1-mediated NEMO deSUMOylation in adipocytes limits inflammatory responses and type-1 diabetes progression. *Nat. Commun.* **6,** 8917 (2015).
